# Use of alcohol and drugs with addiction potential among older women and men in a population-based study. The Nord-Trøndelag Health Study 2006-2008 (HUNT3)

**DOI:** 10.1371/journal.pone.0184428

**Published:** 2017-09-08

**Authors:** Kjerstin Tevik, Geir Selbæk, Knut Engedal, Arnfinn Seim, Steinar Krokstad, Anne-S. Helvik

**Affiliations:** 1 Norwegian National Advisory Unit on Ageing and Health, Vestfold Hospital Trust, Tønsberg, Norway; 2 Department of Public Health and General Practice, Faculty of Medicine, Norwegian University of Science and Technology (NTNU), Trondheim, Norway; 3 Research Centre for Old Age Psychiatric Research, Innlandet Hospital Trust, Ottestad, Norway; 4 Faculty of Medicine, Institute of Health and Society, University of Oslo, Oslo, Norway; 5 Department of Geriatric Medicine, Oslo University Hospital, Oslo, Norway; 6 HUNT Research Centre, Department of Public Health and General Practice, Faculty of Medicine, Norwegian University of Science and Technology (NTNU), Levanger, Norway; 7 Psychiatric Department, Levanger Hospital, Nord-Trøndelag Hospital Trust, Levanger, Norway; 8 St. Olavs University Hospital, Trondheim, Norway; Medizinische Universitat Wien, AUSTRIA

## Abstract

**Background:**

Little is known about the consumption habits of older adults in Norway with respect to alcohol and the use of drugs with addiction potential, such as benzodiazepines, z-hypnotics and opioids, among regular drinkers. We studied the prevalence of self-reported consumption of alcohol on a regular basis in community-living older men and women (≥ 65 years). Furthermore, we investigated the prevalence of dispensed prescribed drugs with addiction potential in older men and women who were regular drinkers.

**Methods:**

We used data from the Nord-Trøndelag Health Study 2006–2008 (HUNT3). Of 12,361 older adults in the HUNT3 study, 11,545 had answered the alcohol consumption item and were included in our study. Regular drinkers were defined as consuming alcohol one or more days a week. Data on dispensed drugs with addiction potential were drawn from the Norwegian Prescription Database. Addiction potential was defined as at least one prescription for benzodiazepines, z-hypnotics or opioids during one year for a minimum of two consecutive years.

**Results:**

In total 28.2% of older Norwegian adults were regular drinkers. Men in the study were more likely to be regular drinkers than women. Drugs with addiction potential were used by 32.4% of participants, and were more commonly used by women. Nearly 12% of participants used benzodiazepines, 19% z-hypnotics and 12.4% opioids. Among regular drinkers, 29% used drugs with addiction potential, which was also more common among women. Adjusted for age, gender and living situation, use of z-hypnotics was associated with regular alcohol intake, while use of opioids was associated with no regular alcohol intake.

**Conclusion:**

The prevalence of the use of drugs with addiction potential was high in a Norwegian population of older adults who reported regular consumption of alcohol. Strategies should be developed to reduce or prevent alcohol consumption among older adults who use drugs with addiction potential.

## Introduction

Although alcohol use tends to decline in old age [1], approximately 40–65% of older adults in the USA [2, 3] and Europe [4, 5] consume alcohol weekly (≥ 1 drink/week). In Finland, the prevalence of older adults who use alcohol weekly (≥ 1 drink/week) ranges from 17% [6] to 75% [7]. These prevalence estimates are affected by differences in the age groups used by different studies. Among older adults, prevalence estimates are also greatly affected by the sample. The estimates can differ between the studied cohorts [2] and differ from a nationally representative sample [3]. Alcohol consumption in recent decades has increased among older adults in the USA [8] and in the Nordic countries [5, 9–11], while in Europe [5] it has been generally stable. As Western populations age, however, it is likely that the number of older adults who consume alcohol and who have alcohol-related problems will increase [5, 12]. Among older adults, those who are younger [9, 13], male [6, 7, 9, 13–15] and not living alone [14] are more likely to consume more alcohol.

Older adults commonly use addictive psychotropic drugs, such as benzodiazepines (BZD) and z-hypnotics, and opioids with addiction potential [3, 16]. In the present study, we use the term “drugs with addiction potential” to describe BZD (hypnotics/sedatives or anxiolytics), z-hypnotics or opioids, and the term “addictive psychotropic drugs” to describe BZD (hypnotics/sedatives or anxiolytics) or z-hypnotics. The prevalence of use of addictive psychotropic drugs has ranged from 5.6% [17] to 8.2% [3] in US studies, while European [18–20] and Nordic studies [6, 21] show prevalences that range from 16% to 31%. In Norway, the proportion of older adults who are receiving prescribed BZD has decreased over the last decade, while the use of BZD-like z-hypnotics has increased slightly among women, but not among men [16]. Although the prevalence differs between countries, opioid use is also quite high among older adults [3, 4, 6, 15, 17]. In the USA, the prevalence of use of opioids (narcotics, analgesics) in older adults has varied from 5.6% [17] to 9.9% [3]. This prevalence is lower in Germany (1–3%) [4, 15], while it may be slightly higher in Finland (10%) [6]. However, since there are no standard criteria for describing the use of drugs with addiction potential, different study criteria may affect the reported prevalence [22]. The observation periods [22] and age groups that are used by different studies also affect the reported prevalence of use. Among older adults, the use of drugs with addiction potential seems to increase with increasing age [16, 23–25] and is associated with female gender [7, 14–16, 23, 24] and living alone [14].

The prevalence of the combined use of alcohol and drugs with addiction potential among older adults is known to be high [26]. This combined use has been studied in older adults in the US [3, 17, 27–29], and in European [4, 19, 20, 23, 30] and Nordic countries [6, 7, 24, 31]. In a large population-based study among US adults older than 57 years (N = 2975) [3], 42% of the participants were regular drinkers (≥ 1 drink/week). Fully 7% of regular drinkers used addictive psychotropic drugs (including antidepressants), and almost 7% combined the use of analgesics with alcohol. A German study of adults older than 60 years (N = 1605) found similar results [4]. Almost 50% of participants consumed alcohol at least once a week, and 7.6% combined use of addictive psychotropic drugs (including antidepressants) and alcohol. The prevalence of combined use of alcohol and drugs with addiction potential was higher in a community-based survey conducted in Finland among adults older than 75 years (N = 521). Thirty-nine percent and 40% of the participants who consumed 1–7 drinks per week also used addictive psychotropic drugs and opioids, respectively [6].

Drugs with addiction potential, such as BZD, z-hypnotics and opioids, interact negatively with alcohol [32], and the associated health risks of this concomitant use are of concern [7]. Aging changes the body’s absorption, distribution and metabolism of alcohol and drugs. Therefore, older adults are susceptible to serious adverse effects from the concomitant use of these substances [30, 32]. Some adverse effects may even occur at low to moderate levels of alcohol consumption (e.g. 1–2 drinks per occasion) [32]. The most common adverse effects are higher blood alcohol levels, altered metabolism of and adverse effects from medication, liver toxicity and sedation [27, 30, 32]. Alcohol enhances the effects of BZD and opioids, such as drowsiness, sedation and decreased motor skills [33]. These adverse effects may further increase the risk of falls, worsen depression, or result in car accidents and death [3, 30, 32, 33]. Even though there has been less examination of BZD-like z-hypnotics (zolpidem), evidence suggests that they act much like BZD in combination with alcohol [34, 35].

Knowledge about the use of alcohol and drugs with addiction potential among older adults is essential to improving preventive strategies that may increase quality of life, reduce mortality and lower health care costs [3, 12, 28, 36]. In Norway, we know very little about the consumption of alcohol in older adults [9, 21] and the combined use of alcohol and drugs with addiction potential [21]. Almost all previous international studies have either used self-reporting for the use of drugs with addiction potential [7, 15, 19, 24, 27, 28, 31] or participants have brought their prescription sheets and medication containers to the interview [3, 4, 6, 17, 20, 23, 30]. One study used prescription claims data [29]. In Norway, a nationwide prescription database [37] can be linked to population-based health studies of older Norwegian adults. This is likely to provide a more precise estimate of drugs with addiction potential taken in combination with self-reported consumption of alcohol.

The main aim of this study was to investigate the self-reported prevalence of regular alcohol consumption in community-living older men and women aged 65 years or older, and to examine the prevalence of dispensed prescribed drugs with addiction potential in older men and women who regularly drink alcohol.

## Materials and methods

### Study setting and data sources

Data from the third wave of the Nord-Trøndelag Health Study (HUNT3) were used. The third cross-sectional HUNT Survey was completed between October 2006 and June 2008 in Nord-Trøndelag County, Norway, which had a population of 128,694 in 2006. Every resident who was 20 years or older (N = 93,860) was invited to participate in the study and roughly 50,000 (54% of those invited) chose to do so. Full details of the HUNT study have been described elsewhere [38].

The data for this study relied on information from all community-dwelling individuals ≥ 65 years who answered one item about alcohol consumption. Of 12,361 individuals ≥ 65 years, 816 (7%) did not answer this item, which meant our total sample consisted of 11,545 individuals. The participation rate in HUNT3 declined by age, from 71.1% in age group 60–69 years to 17.2% in age group 90 + [38].

Previous comparisons of those who participated and did not participate in HUNT3 have shown that poor people in need of social support participate to a lesser extent than others. Among these, there will probably be people with serious addictions. Furthermore, it has been shown that the highest non-participation rate among the oldest individuals is in people with chronic diseases. This may suggest that our study primarily evaluates those without the most serious alcohol or drug problems and those who can come to an examination station [39].

Data on dispensed prescribed drugs with addiction potential were drawn from the Norwegian Prescription Database (NorPD) at the Norwegian Institute of Public Health from 2005 to 2009 for HUNT3 participants. All pharmacies in Norway have been legally obliged to send in electronic data to the NorPD on all prescriptions starting 1 January 2004 [37].

### Measures

#### Alcohol consumption

Alcohol consumption was rated by asking participants how often they drank alcohol in the previous 12 months. The response options were as follows: 1 = 4–7 times a week, 2 = 2–3 times a week, 3 = about once a week, 4 = 2–3 times a month, 5 = about once a month, 6 = a few times a year, 7 = not in the last year and 8 = never drink alcohol.

Regular consumption of alcohol was defined as weekly intake (regular drinkers = intake of alcohol one or more days a week, including response categories 1 to 3) [3, 4]. Regular drinkers were further divided into three categories based on drinking frequency per week: 1, 2–3 and ≥ 4 days with alcohol intake per week.

#### Drugs with addiction potential

We chose to focus on BZD, z-hypnotics and opioids for the present study as drugs with addiction potential. The drugs were coded according to the World Health Organization Anatomical Therapeutic Chemical (ATC) classification system [40]. BZD was defined by ATC-code N03AE (antiepileptic), N05BA (anxiolytic) and N05CD (hypnotic and sedative) [35, 40]. Z-hypnotics were defined by ATC-code N05CF [35, 40] and opioids by ATC-code N02A [40].

In line with previous studies, we defined the use of drugs with addiction potential as at least one prescription of BZD, z-hypnotics or opioids within one year for a minimum of two consecutive years (2005/2006, 2006/2007, 2007/2008 or 2008/2009) [21, 25, 41, 42].

#### Sociodemographic variables

Socio-demographic information about age, gender and living situation was collected from HUNT3.

### Ethics and data protection

HUNT3 Survey participants signed an informed consent form allowing use of their data for future medical research. HUNT research is carried out in accordance with the guidelines of the Regional Committee of Medical Research Ethics (REC) and the Norwegian Data Inspectorate Authority and applicable law [43]. REC, the Norwegian Social Science Data Services, the Norwegian Data Inspectorate Authority and the Norwegian Institute of Public Health have all approved the present study.

The final linkage between data from HUNT3 and the NorPD was undertaken by NorPD. All personal identification was subsequently removed to ensure anonymity according to Norwegian regulations for linkage of health registers.

### Statistics

The data were analyzed with SPSS, version 23. Descriptive statistics were used to estimate the prevalence of regular alcohol consumption, use of drugs with addiction potential, and use of drugs with addiction potential in older adults who drank alcohol on a regular basis. Categorical data were analyzed by Chi-Square test. Age, which is the only continuous descriptive variable and not normally distributed, was analyzed by the Mann-Whitney U Test or the Kruskal Wallis test.

We used binary logistic regression analyses (the Enter method) to investigate the association between the outcome measure “regular drinker” (≥ 1 day/week versus not) and “use of drugs with addiction potential” (exposure). Possible confounding was evaluated using Directed Acyclic Graphs (DAG) [44]. Age [9, 13, 16, 23–25], gender [6, 7, 9, 13–16, 23, 24] and living situation [14] are associated with both the outcome measure “regular drinker” and the exposure variable “drugs with addiction potential.” We considered these variables to be possible confounders, so we adjusted for this in our analyses. Independent variables in the analyses were therefore as follows: age, gender, living situation (living alone or not) and use of BZD (yes/no), z-hypnotics (yes/no) and opioids (yes/no). Age was linearly associated with the outcome and thus age could be used as a continuous variable in the adjusted analysis. The unadjusted and adjusted analyses were presented with odds ratios (OR) and 95% confidence intervals (CI). Results were considered significant when p < 0.05.

## Results

The demographics characteristics of the participants (N = 11,545) are described in Table 1. The participants’ mean age was 73.7 (SD = 6.3); 52.7% were female.

**Table 1:** Overall sample characteristics and drinking status in older Norwegian adults (≥ 65 years, N = 11,545). The HUNT Study 2006–08 (HUNT3).

		Overall	Non-regular drinkers	Regular drinkers	Regular drinkers: drinking frequency per week		
			(< 1 day/week)	(≥ 1 day/week)	1 day	2–3 days	≥ 4 days
Overall	N (%)	11545 (100)	8286 (71.8)	3259 (28.2)	1705 (52.3)	1187 (36.4)	367 (11.3)
Age	Median (range)	72.70 (65–100.8)	73.60 (65–100.8)	70.6 (65–97.7)^a^***	70.7 (65–92.5)	70.5 (65–97.7)	71.2 (65–96.9)^b^^NS^
Age							
65–69 years	N (%)	4032 (34.9)	2554 (30.8)	1478 (45.4)^c^***	764 (44.8)	556 (46.8)	158 (43.1)^d^^NS^
70–74 years	N (%	3056 (26.5)	2150 (25.9)	906 (27.8)	466 (27.3)	338 (28.5)	102 (27.8)
≥ 75 years	N (%)	4457 (38.6)	3582 (43.3)	875 (26.8)	475 (27.9)	293 (24.7)	107 (29.1)
Gender							
Male	N (%)	5461 (47.3)	3476 (42.0)	1985 (60.9)^c^***	1007 (59.1)	739 (62.3)	239 (65.1)^d^*
Female	N (%)	6084 (52.7)	4810 (58.0)	1274 (39.1)	698 (40.9)	448 (37.7)	128 (34.9)
Living alone	N (%)	2997 (26.0)	2454 (29.6)	543 (16.7)^c^***	302 (17.7)	448 (37.7)	128 (34.9)
BZD, z-hypnotics or opioids^1^	N (%)	3741 (32.4)	2784 (33.6)	957 (29.4)^c^***	488 (28.6)	345 (29.1)	124 (33.8)^d^^NS^

BZD = benzodiazepines

^1^At least one prescription of BZD, z-hypnotics or opioids in one year for a minimum of two consecutive years (2005/2006, 2006/2007, 2007/2008 or 2008/2009). BZD defined by N03AE, N05BA and N05CD. Z-hypnotics defined by N05CF. Opioids defined by N02A.

^a^Significance testing with Mann Whitney U test between regular (≥ 1 day/week) and non-regular drinkers (< 1 day/week)

^b^Significance testing with Kruskal-Wallis test between drinking frequency (1, 2–3 or ≥ 4 days per week) among regular drinkers (≥ 1 day/week)

^c^Significance testing with Chi-square test between regular (≥ 1 day/week) and non-regular drinkers (< 1 day/week)

^d^Significance testing with Chi-square test between drinking frequency (1, 2–3 or ≥ 4 days per week) among regular drinkers (≥ 1 day/week)

*P < 0.05

***P < 0.001

NS = non-significant

### Consumption of alcohol and use of drugs with addiction potential

In total, 28.2% (3,259/11,545) of older Norwegian adults (≥ 65 years) were regular drinkers of alcohol according to our definition (≥ 1 day/week) (Table 1). Among regular drinkers more than half (52.3%) reported drinking once a week, about one-third (36.4%) reported drinking two to three days per week, while about 11% consumed alcohol between four to seven days per week. Regular drinkers were more often men than women (60.9% versus 39.1%, p < 0.001), younger than non-regular drinkers (median 70.6 years versus 73.6 years, p < 0.001) and more often living with someone.

Nearly one-third (32.4%) of participants used drugs with addiction potential (41.4% women and 22.8% men, p < 0.001). Almost 12% of the participants used BZD, 19% z-hypnotics and 12.4% opioids.

### Drugs with addiction potential in relation to drinking

Table 2 shows the prevalence of the use of drugs with addiction potential according to drinking status and drinking frequency. The prevalence of the use of drugs with addiction potential among regular drinkers increased slightly with increasing drinking frequency per week, but the difference was not significant. The use of drugs with addiction potential was significantly more common among women, in both non-regular drinkers and regular drinkers. Regular drinkers were significantly less likely to use drugs with addiction potential compared to non-regular drinkers.

**Table 2:** Prevalence of use of benzodiazepines, z-hypnotics and opioids according to drinking status and drinking frequency in older Norwegian adults (≥ 65 years, N = 11,545). The HUNT Study 2006–08 (HUNT3).

		Non-regular drinkers: < 1 day/week			Regular drinkers: ≥ 1 day/week			Regular drinkers: Drinking frequency per week		
		Total	Female	Male	Total	Female	Male	1 day	2–3 days	4 ≥ days
		N = 8286			N = 3259			n = 1705	n = 1187	n = 367
BZD^1^	N (%)	1021 (12.3)^a^**	780 (16.2)	241 (6.9)^b^***	344 (10.6)	197 (15.5)	147 (7.4)^c^***	173 (10.1)	133 (11.2)	38 (10.4)^d^^NS^
Z-hypnotics^2^	N (%)	1611 (19.4)^a^*	1209 (25.1)	402 (11.6) ^b^***	580 (17.8)	333 (26.1)	247 (12.4)^c^***	301 (17.7)	206 (17.4)	73 (19.9)^d^^NS^
Opioids^3^	N (%)	1111 (13.4)^a^***	744 (15.5)	367 (10.6) ^b^***	322 (9.9)	148 (11.6)	174 (8.8)^c^**	159 (9.3)	121 (10.2)	42 (11.4)^d^^NS^
BZD or z-hypnotics	N (%)	2237 (27.0)^a^**	1680 (34.9)	557 (16.0) ^b^***	793 (24.3)	448 (35.2)	345 (17.4) ^c^***	410 (24.0)	284 (23.9)	99 (27.0)^d^^NS^
BZD, z-hypnotics or opioids	N (%)	2784 (33.6)^a^***	1993 (41.4)	791 (22.8) ^b^***	957 (29.4)	505 (39.6)	452 (22.8) ^c^***	488 (28.6)	345 (29.1)	124 (33.8)^d^^NS^

BZD = benzodiazepines

^1^At least one prescription of BZD in one year for a minimum of two consecutive years (2005/2006, 2006/2007, 2007/2008 or 2008/2009). BZD defined by N03AE, N05BA and N05CD.

^2^At least one prescription of z-hypnotics in one year for a minimum of two consecutive years (2005/2006, 2006/2007, 2007/2008 or 2008/2009). Z-hypnotics defined by N05CF.

^3^At least one prescription of opioids in one year for a minimum of two consecutive years (2005/2006, 2006/2007, 2007/2008 or 2008/2009). Opioids defined by N02A.

^a^ Significance testing using Chi-square test between regular drinkers (≥ 1 day/week) and non-regular drinkers (< 1 day/week)

^b^ Significance testing using Chi-square test between female and male among non-regular drinkers (< 1 day/week)

^c^ Significance testing using Chi-square test between female and male among regular drinkers (≥ 1 day/week)

^d^ Significance testing using Chi-square test between drinking frequency (1, 2–3 or ≥ 4 days per week) among regular drinkers (≥ 1 day/week)

*P *<* 0.05

**P *<* 0.01

***P < 0.001

NS = non-significant

In logistic regression analyses adjusted for age, gender and living situation, users of z-hypnotics were more likely to be regular drinkers while users of opioids were less likely to be regular drinkers (Table 3).

**Table 3:** Association between use of drugs with addiction potential and regular drinking (≥ 1 day/week) among older Norwegian adults (≥ 65 years, N = 11,545) in unadjusted and adjusted logistic regression analyses. The HUNT Study 2006–08 (HUNT3).

	Regular drinkers (≥ 1 day/week) versus no regular drinkers			Regular drinkers (≥ 1 day/week) versus no regular drinkers		
	Unadjusted^a^ LRA			Adjusted^b^ LRA		
	OR	95% CI	P	OR	95% CI	P
**Demographics**						
Age (continuous)	0.95	0.93–0.94	< 0.001	0.94	0.93–0.95	< 0.001
Gender (men)	2.16	1.99–2.34	< 0.001	2.07	1.90–2.26	< 0.001
Living alone	0.48	0.43–0.53	< 0.001	0.67	0.60–0.75	< 0.001
**Use of drugs with addiction potential**						
BZD^1^	0.84	0.74–0.96	0.008	1.12	0.97–1.29	0.114
Z-hypnotics^2^	0.90	0.81–1.00	0.042	1.29	1.15–1.44	< 0.001
Opioids^3^	0.71	0.62–0.81	< 0.001	0.76	0.66–0.87	< 0.001

LRA = logistic regression analyses; OR = odds ratio; CI = confidence interval; BZD = benzodiazepines

^1^At least one prescription of BZD in one year for a minimum of two consecutive years (2005/2006, 2006/2007, 2007/2008 or 2008/2009). BZD defined by N03AE, N05BA and N05CD.

^2^At least one prescription of z-hypnotics in one year for a minimum of two consecutive years (2005/2006, 2006/2007, 2007/2008 or 2008/2009). Z-hypnotics defined by N05CF.

^3^At least one prescription of opioids in one year for a minimum of two consecutive years (2005/2006, 2006/2007, 2007/2008 or 2008/2009). Opioids defined by N02A.

^a^Unadjusted binary logistic regression analysis. Independent variables included separately. Dependent variable: Regular drinkers (< 1 day per week reference category). Independent variables: Age (continuous variable), gender (female reference category), living alone (not living alone reference category), BZD (not using BZD reference category), z-hypnotics (not using z-hypnotics reference category), opioids (not using opioids reference category).

^b^Adjusted binary logistic regression analysis. All variables included in the unadjusted analyzes were used.

## Discussion

In this large population-based HUNT study (HUNT3, 2006–08) among community-living men and women 65 years or older in Norway, 28% were regular drinkers according to our definition. The prevalence of regular drinking was higher among men. The overall prevalence of prescribed drugs with addiction potential was around 30% and was more prevalent among women. Almost 30% of regular drinkers used drugs with addiction potential, which was also more common among women. In adjusted analyses, the use of z-hypnotics was associated with regular alcohol intake, while use of opioids was associated with no regular alcohol intake.

### Alcohol consumption and use of drugs with addiction potential

In our study, 28% of older adults consumed alcohol weekly, while about 40 to 75% of older adults in the USA, Germany and Finland were regular drinkers [3, 4, 7]. Thus, older Norwegian adults seem to consume alcohol weekly less often than their counterparts in the USA and other European countries. Historically, Norway has had a strict alcohol policy with high prices and restricted availability [9, 45], which may partly explain these differences. Our finding that more men were regular drinkers (≥ 1 day/week) compared to women is, however, consistent with prior reports [2, 3, 7]. In our study, alcohol consumption was measured as “drinking days per week,” while others have used “drinks per week,” [3] “grams of alcohol per day,” [4] “times per week,” [7] and “units of alcohol” [7] as measures. These differences may complicate the comparison between studies and countries [5]. Furthermore, the comparison between the cited studies in the USA, Germany and Finland may be controversial since the participants had somewhat different age profiles [3, 4, 6, 7].

Our data suggest that use of drugs with addiction potential is common among older adults in Norway, i.e. 12% used BZD, 19% z-hypnotics and 12.4% opioids, respectively. These results are consistent with data from NorPD with respect to use of BZD and z-hypnotics among adults 65 years or older in the Norwegian population in general [16]. However, participants in HUNT3 used opioids less often than adults 65 years or older in the general Norwegian population [16]. The non-participation rate among older adults with chronic diseases was high in HUNT3, and especially high among older women aged 60–79 years with cancer, osteoporosis, fibromyalgia and arthroses [39]. Since opioids are used in the treatment of chronic malignant and non-malignant pain [46], the lower prevalence of opioid use in our sample seems reasonable and caused by selection.

Compared to international studies, the proportion of BZD users in the present study seems to be higher than among older adults in the USA [3, 17], but lower than among older adults in France [18, 20], Spain [19] and Finland [6]. The use of z-hypnotics is not specified in these studies [3, 6, 17–20], and therefore has not been compared to our results of use of z-hypnotics. Use of opioids in our sample is quite consistent with use among older adults in the USA [3] and Finland [6], but is higher than among older adults in Germany [4, 15]. In line with other studies [15, 18], the prevalence of use of BZD and opioids was more pronounced among women than men.

A direct comparison of the use of drugs with addiction potential between countries is challenging due to differences in the sample characteristics of the various published studies [6, 15], but also because of differences in how drug use is defined [15, 19] and because of differences in data collection methods, i.e. use of a prescription database [37], self-report [19] or by examination of medication containers or prescriptions [3, 6, 17, 18, 20]. We believe that the use of a national prescription database (NorPD) [37] is a reliable resource for data collection on the use of drugs with addiction potential.

### Use of drugs with addiction potential in participants who drink alcohol regularly

In our study, the use of addictive psychotropic drugs and opioids with addiction potential in participants who drank alcohol regularly was 24.3% and 9.9% respectively. Thus, the use of addictive psychotropic drugs in Norwegian older adults who consumed alcohol on a regular basis was higher compared to two large community-based studies conducted in the USA [3] and Germany [4], where the prevalence of combined use (including antidepressants) was about 7% in both countries. On the other hand, in the nearby country of Finland, the use of addictive psychotropic drugs and opioids was higher among older adults who consumed alcohol weekly, 39% and 40% respectively [6]. However, as already discussed, due to differences in sample characteristics and methodologies between studies, a comparison of the prevalence of use of drugs with addiction potential among older adults who consume alcohol is difficult.

The prevalence of use of drugs with addiction potential among regular drinkers and non-regular drinkers was higher among women than men in our study. This result is supported by the findings from another study [15], where the prevalence of addictive psychotropic drug use was more prevalent in both risk drinker and non-risk drinker older women compared to men. Risk drinking was defined in this circumstance as drinking more than 20 grams of pure alcohol per day for women and more than 30 grams pure alcohol per day for men [15]. However, our result is in contrast to results from another study from Finland [7] where the use of addictive psychotropic drugs in individuals who consumed alcohol regularly was higher among men than women.

Although our finding was not statistically significant, it is notable that we found the prevalence of the use of drugs with addiction potential among regular drinkers increased slightly with the drinking frequency per week. Moreover, in adjusted analyses, users of z-hypnotics were more likely to be regular drinkers than non-regular drinkers. Given that very small doses of alcohol can cause adverse alcohol and drug interaction in combined use with sedative medication [29, 33], the findings may suggest that many older adults in this Norwegian population are at risk for adverse effects. However, an encouraging finding in the adjusted analyses was that users of opioids were significantly less likely to be regular drinkers. Opioid use is associated with malignant and non-malignant chronic pain [46], so that the addition of a strong stimulant like alcohol may deter its use in older adults who take opioids to treat chronic pain [47].

The relatively high prevalence of the use of drugs with addiction potential among regular drinkers (≥ 1 day/week) in our study does not provide proof that there is extensive simultaneous use of alcohol and drugs with addiction potential, however [17]. Neither our or previous studies [3, 4, 6, 7, 17, 19, 20, 23, 24, 27–31] had accurate time-line information about the consumption of alcohol and drugs with addiction potential. This means that our study does not provide information on the combined use of alcohol and drugs with addiction potential. It is possible that participants did not consume alcohol while taking drugs with addiction potential. The interaction of alcohol and drugs with addiction potential may only occur if both substances are present in the body simultaneously [17]. Regular drinkers may not drink on the day they take drugs with addiction potential, or these drugs may not be taken daily [17]. Thus, the prevalence of the simultaneous use of alcohol and drugs with addiction potential has yet to be studied.

Nevertheless, our results have important clinical implications. Strategies should be developed to prevent the combined use of alcohol and drugs with addiction potential in older adults [3]. Although we were unable to investigate the reasons behind possible combined use, previous research has suggested that many older adults are often unaware of the potential harm of concomitant use [30, 48]. This may be due to the fact that health personnel are less likely to provide this type of information to older adults, including restrictions on alcohol consumption when using certain drugs [49, 50]. A study from the USA reported that only 19% of patients aged 60 and older had been asked about the use of alcohol and drugs by their general medical provider (e.g. primary care physician and nurse) in the past 12 months [50]. In Norway, it has been reported that health personnel find it difficult to talk about alcohol consumption with older adults, because it is considered a private matter, and furthermore, the health personnel often did not have routines for assessing alcohol consumption [51, 52]. There is a need to improve both legitimacy and routines of addressing alcohol consumption in clinical practice [51, 52], especially in older adults who take drugs with addiction potential. Older adults’ drinking behavior needs to be reviewed in order to identify patients at high risk [30] and to avoid adverse effects. Information provided by physicians about adverse effects from the simultaneous consumption of alcohol and certain drugs may decrease alcohol drinking among older adults [53, 54]. Health personnel, specifically physicians, nurses and pharmacists, are in a position to counsel older adults on the safe use of alcohol and drugs and to encourage patients to avoid alcohol consumption while taking drugs with addiction potential [30, 55].

### Strengths and limitations

Our study has several strengths. Firstly, the data come from a large population-based study [43], which gives us a big enough sample size to allow for more precise estimates [17]. Furthermore, Nord-Trøndelag County, where the study was undertaken, is considered fairly representative of Norway based on geography, economy, industry, sources of income, age distribution, morbidity and mortality [39, 56]. However, the county does not contain any large cities [9], and the results are probably not generalizable to such areas. The level of education and income is a little below the Norwegian average [56]. Since a higher level of education [57, 58], higher income [57, 58] and living in urban areas [59, 60] are positively associated with alcohol consumption, this may have affected the prevalence of regular drinking in our study. Secondly, the nationwide prescription database (NorPD) with prescriptions records of BZD, z-hypnotics and opioids [37] were linked to data from HUNT3, through the unique Norwegian 11-digit personal identification number. Thus, we avoid recall bias of drug use [26, 61], even if the consumption of alcohol was self-reported and may be underestimated, especially among heavy drinkers [13, 62, 63]. The underestimation of alcohol consumption among older adults may also be due to memory impairment, the stigma associated with drinking [6, 13] and self-medication using alcohol, which is not always reported as alcohol consumption in alcohol surveys [64].

The study also has some notable limitations. Firstly, the participation rate in HUNT3 declined considerably with increasing age [38]. Non-participation in health surveys has been found to be higher among individuals with substance use disorders (use of alcohol, opioids, sedative, hypnotics or anxiolytics) and mental disorders [65, 66]. Thus, it is likely that participants with high alcohol consumption and medication use did not participate in the present study. Furthermore, some participants in HUNT3 were excluded (7%) due to a missing answer on the item concerning alcohol consumption, and we do not know whether those with a missing answer were more likely than responders to drink alcohol regularly or use drugs with addiction potential. However, there was no difference in HUNT3 in alcohol drinking (i.e. drinking 2–3 times a week or more often) between responding and non-responding men older than 60, or between women aged 60–79 years [39]. Women older than 80 who responded to the survey drank more often than non-responding women [39]. Secondly, alcohol consumption was measured by drinking days per week, and our study does not provide information concerning the amount of alcohol consumed. This could lead to false assumptions, as it is unclear if someone who drinks 4 days/week consumes more alcohol than someone who drinks 2 days/week. Thirdly, the data from NorPD does not take into account whether or not the participants have taken the drugs they were prescribed [26], and we do not know the exact criteria that physicians use when they prescribe drugs with addiction potential [67]. Further, it is not possible to derive actual information concerning the duration of drug use, and it is believable that some participants did not use them on a regular basis, but rather in during specific circumstances, such as after surgery.

## Conclusions

Our study found that fully one-third of community-living adults in Norway aged 65 years or older were regular drinkers (≥ 1 day/week). About 30% used drugs with addiction potential (BZD, z-hypnotics or opioids). The prevalence of the use of drugs with addiction potential was almost 30% in older adults who reported regularly consuming alcohol (≥ 1 day/week). This study does not provide information on the combined use of alcohol and drugs with addiction potential. It is possible that participants did not consume alcohol while they were taking medication. Nevertheless, our study indicates that many older Norwegian adults may be at risk for adverse effects since very small doses of alcohol can cause adverse effects when combined with the use of drugs with addiction potential. Our results have important clinical implications, and strategies should be developed to better reduce or prevent alcohol consumption among older adults who use drugs with addiction potential.
